# Synthesis, Crystal Structure, Spectra and Quantum Chemical Study on 1-Phenyl-3-(4-nitrophenyl)-5-(2-thienyl)-2-pyrazoline

**DOI:** 10.3390/molecules19045313

**Published:** 2014-04-23

**Authors:** Huan-Mei Guo, Pu-Su Zhao, Qian Wu, Yu-Feng Li

**Affiliations:** 1 Microscale Science Institute, Weifang College, Weifang 261061, China; E-Mails: wuqianwq2006@163.com (Q.W.); liyufeng8111@163.com (Y.-F.L.); 2 Jiangsu Key Laboratory for Chemistry of Low-Dimensional Materials, Huaiyin Normal University, Huaian 223300, China; E-Mail: zhaopusu@hytc.edu.cn

**Keywords:** synthesis, crystal structure, spectra, DFT, second order optical nonlinearity

## Abstract

1-Phenyl-3-(4-nitrophenyl)-5-(2-thienyl)-2-pyrazoline was synthesized and characterized by elemental analysis, IR and X-ray single crystal diffraction. UV-Vis spectra and fluorescence spectra were measured. Density functional theory calculations on the structure of the title compound were performed at the B3LYP/6-311G** level of theory. NPA atomic charge distributions indicate that, although the S atom in the thienyl ring loses coordination capacity, the title compound still may be used as a potential multi-dentate ligand to coordinate with metallic ions. The calculation of the second order optical nonlinearity was carried out. Natural bond orbital analyses indicate that the electronic absorption bands are mainly derived from the contribution of *n* → π* and *π* → π* transitions. Fluorescence spectra determination shows that the title compound is a potential orange-light emitting material.

## 1. Introduction

Pyrazoline derivatives are five-membered, nitrogen-containing heterocyclic compounds with high hole-transport efficiency, excellent blue emission and high quantum yield [1,2,3], which have made them useful as fluorescent brightening agents, fluorescence chemosensors, hole-transport materials in electrophotography, OLED and as fluorescent materials [4,5,6,7,8]. Since 2001, different groups have prepared pyrazoline nanoparticles ranging from tens to hundreds of nanometers in size by using the re-precipitation method and explored their size-tunable optical properties for application in optoelectronic devices [9,10,11]. Our group has also reported the synthesis, structure and spectra properties of a series of 2-pyrazolines by experimental and theoretical methods [12,13,14,15,16,17,18].

Among numerous pyrazoline derivatives, one type of pyrazolines containing thienyl groups has been proved very important. For example, they can be introduced at the C-4 position of a 1,8-naphthalimide derivative and lead to stronger fluorescence emission [19]. Various synthetic methods for this type of pyrazolines have been reported, such as a microwave method [20], a catalytic method in the present of base [21] a refluxing method [22], and so on.

On the other hand, with the development of theoretical chemistry, density functional theory (DFT) has become an increasingly useful tool to compare and verify experimental studies. The success of DFT is mainly due to the fact that it describes small molecules more reliably than Hartree-Fock theory. It is also computationally more economic than wave function based methods with inclusion of electron correlation [23,24].

**Scheme 1:**
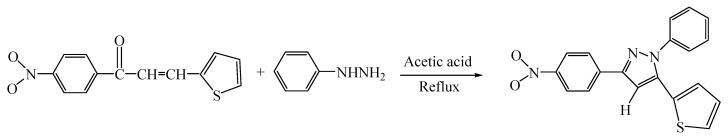
Synthetic route to the title compound.

However, to our knowledge, no structural data obtained either by experimental or theoretical methods have been reported so far for the title compound, 1-phenyl-3-(4-nitrophenyl)-5-(2-thienyl)-2-pyrazoline, which contains both a pyrazolinyl and a substituted thienyl group. Thus, in order to characterize the correlation between molecular structure and macroscopic properties in the studied compound, it seemed essential to undertake a detailed comparative study of the isolated molecule and the solid state unit. In this paper, after the title compound was synthesized by a refluxing method (see Scheme 1), a concerted approach by X-ray crystallography and DFT calculations was used, which takes advantage of both the high interpretative power of the theoretical studies and the precision and reliability of the experimental method. We hope that the research presented herein will be helpful for the design of pyrazoline-based electroluminescent devices and materials.

## 2. Results and Discussion

### 2.1. Description of the Crystal Structure

**Figure 1:**
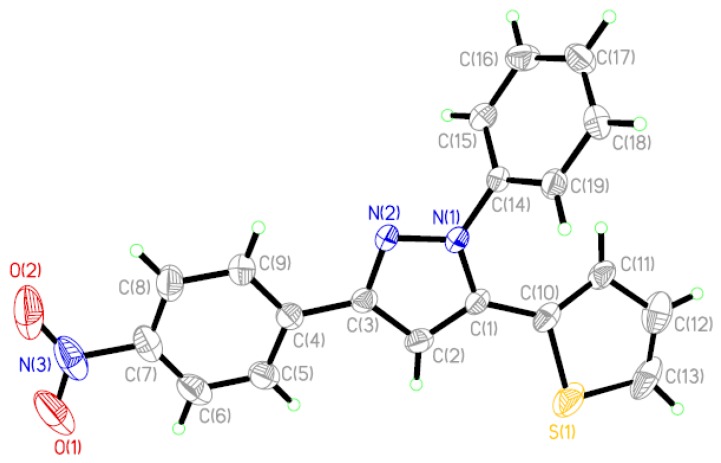
Molecular structure with the atomic numbering scheme for the title compound.

**Figure 2:**
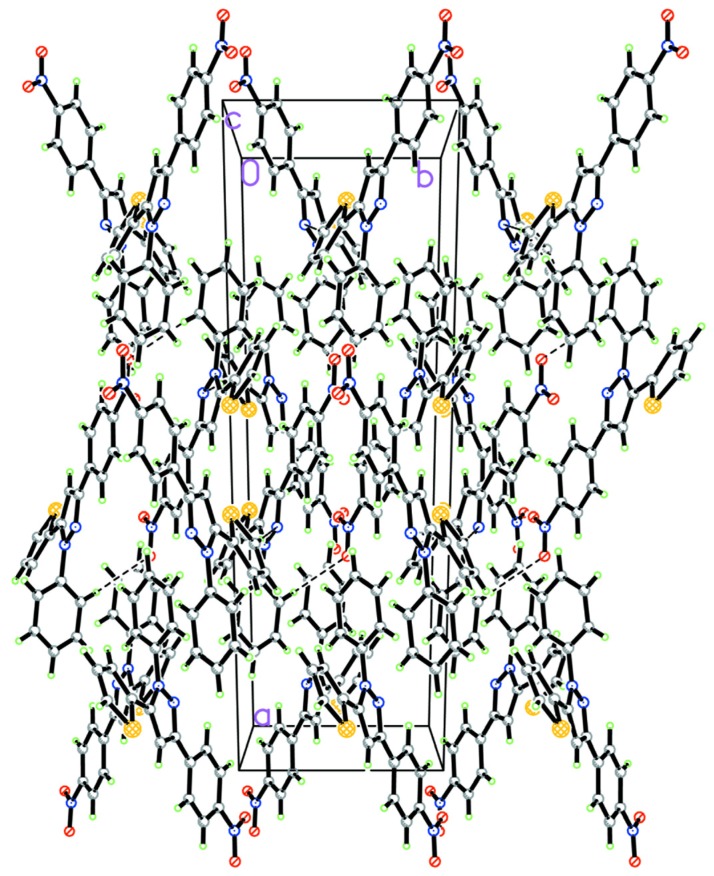
A view of the crystal packing down the *c* axis for the title compound.

The displacement ellipsoid plot with the numbering scheme for the title compound is shown in Figure 1. Figure 2 shows a perspective view of the crystal packing in the unit cell. Selected bond lengths and bond angles by X-ray diffractions are listed in Table 1 along with the calculated bond parameters.

The molecular structure of the title compound consists of discrete [PhC_3_HN_2_PhNO_2_C_4_H_3_S] entities. All of the bond lengths and bond angles in the phenyl rings are in the normal range. In the pyrazolinyl ring of the molecule, the C=N bond length [1.332(4) Å] is longer than that in the similar structure [C=N 1.297(2) Å] [25]. The bond lengths of C–N [1.365(4) Å] and N–N [1.356(3) Å] are shorter than those found in the above-cited structure [C–N 1.474(2) Å, N–N 1.380(2) Å] [25]. The pyrazolinyl ring makes dihedral angles of 5.39(3), 60.10(3) and 19.27(11), respectively, with the *p*-nitrophenyl, phenyl and 2-thienyl rings.

**Table 1:** Selected structural parameters by X-ray and theoretical calculations.

Bond Lengths (Å)	Experiment	B3LYP/6-311G**	Bond Lengths (Å)	Experiment	B3LYP/6-311G**
S(1)–C(13)	1.675(5)	1.730	C(1)–C(10)	1.458(4)	1.457
S(1)–C(10)	1.704(3)	1.750	C(2)–C(3)	1.402(4)	1.415
O(1)–N(3)	1.201(6)	1.225	C(3)–C(4)	1.466(4)	1.468
O(2)–N(3)	1.225(6)	1.225	C(4)–C(5)	1.389(5)	1.403
N(1)–N(2)	1.356(3)	1.348	C(4)–C(9)	1.393(5)	1.405
N(1)–C(1)	1.365(4)	1.380	C(5)–C(6)	1.381(5)	1.387
N(1)–C(14)	1.430(4)	1.429	C(10)–C(11)	1.402(5)	1.373
N(2)–C(3)	1.332(4)	1.334	C(14)–C(19)	1.373(4)	1.394
N(3)–C(7)	1.480(5)	1.474	C(15)–C(16)	1.381(5)	1.391
C(1)–C(2)	1.374(4)	1.383	C(17)–C(18)	1.362(5)	1.394
**Bond Angles (°)**			**Bond Angles (°)**		
C(13)–S(1)–C(10)	92.7(2)	91.6	C(8)–C(7)–C(6)	121.6(4)	121.6
N(2)–N(1)–C(1)	112.0(2)	111.9	C(11)–C(10)–S(1)	109.6(2)	110.5
C(3)–N(2)–N(1)	105.4(2)	106.0	C(10)–C(11)–C(12)	111.4(4)	112.9
O(1)–N(3)–O(2)	125.1(5)	124.6	C(13)–C(12)–C(11)	113.4(4)	113.3
N(1)–C(1)–C(2)	105.8(2)	105.8	C(12)–C(13)–S(1)	112.9(3)	111.7
C(1)–C(2)–C(3)	106.2(3)	105.7	C(19)–C(14)–C(15)	121.0(3)	120.6
N(2)–C(3)–C(2)	110.6(3)	110.6	C(17)–C(16)–C(15)	120.5(3)	120.3
C(5)–C(4)–C(9)	117.9(3)	118.7	C(17)–C(18)–C(19)	119.9(3)	120.4

In the crystal lattice, there are two intermolecular interactions (C–H···Y, Y=N) [26,27] and some C–H···*π* supramolecular interactions (see Table 2) [28]. In the solid state, all above supramolecular interactions stabilize the crystal structures.

**Table 2:** Hydrogen bonds and C–H···*π* supramolecular interactions *^a^*.

D–H···A	Symmetry	H···A (Å)	D···A (Å)	∠D–H···A (°)
C(13)–H(13)···N(2)	x, 1 − y, −1/2 + z	2.510(3)	3.439(3)	176.98
C(19)–H(19)···O(1)	−x, −y, −z	2.552(2)	3.279(1)	135.30
C(11)–H(11)···Cg(4)	x, y, z	3.037(1)	3.743(1)	134
C(16)–H(16)···Cg(4)	1/2 − x, 1/2 + y, 1/2 − z	3.209(2)	3.879(3)	131
C(18)–H(18)···Cg(1)	1/2 − x, 1/2 − y, −z	2.809(2)	3.596(2)	143

*^a^* Cg(1) and Cg(4) denote thienyl ring and phenyl ring C(14)–C(19), respectively.

### 2.2. Optimized Geometry

DFT calculations were performed on the title compound at B3LYP/6–311G** level of theory. Some optimized geometric parameters are also listed in Table 1. In view of the bond lengths in Table 1, most predicted values are longer than experimental ones and the biggest difference between the theoretical and experimental values occurs at S(1)–C(13) bond, with the different values being 0.0554 Å. As for the bond angles, most predicted values correspond with the experimental values and the biggest difference is seen in the bond angle of C(12)–C(13)–S(1), with a difference between values of 1.22°. The reasons for the above discrepances maybe as follows: (1) the theoretical values correspond to the isolated molecule in the gas-phase and the experimental values are from the molecule in the solid state. The geometry of the solid-state structures is subject to intermolecular forces, such as van der Waals interactions and crystal packing forces, which make most of the experimental bond lengths shorter than the theoretical ones; (2) in the solid state, there exists a C–H···*π* supramolecular interaction corresponding with the thienyl ring (see Table 2), while in theoretical calculations, supramolecular interactions are neglected, which, to some extent, may lead to bigger bond length and bond angle differences between the experiments and calculations corresponding to the thienyl ring. Despite of some differences, the DFT method used here can reproduce the molecular geometry on the whole and it is the basis for our following discussion.

### 2.3. Atomic Charge Distributions

Based on B3LYP/6-311G** optimized geometry, NPA atomic charge distributions of the title compound were calculated. All of the atomic charges of non-hydrogen atoms are listed in Table 3.

**Table 3:** NPA atomic charge distributions obtained at B3LYP/6-311G** level.

Atom	Charges (*e*)	Atom	Charges (*e*)	Atom	Charges (*e*)
N(1)	−0.16920	C(4)	−0.03329	C(11)	−0.22313
N(2)	−0.28191	C(5)	−0.18105	C(12)	−0.24353
C(1)	0.13219	C(6)	−0.17616	C(13)	−0.37017
C(2)	−0.27626	C(7)	0.05712	C(14)	0.14793
C(3)	0.13913	C(8)	−0.17632	C(15)	−0.19406
N(3)	0.51394	C(9)	−0.16957	C(16)	−0.18771
O(1)	−0.38790	S(1)	0.41951	C(17)	−0.19584
O(2)	−0.38977	C(10)	−0.22371	C(18)	−0.18649
				C(19)	−0.20411

As seen from Table 3, all non-hydrogen atomic charge distributions are not only influenced by the atomic electronegativity, but also by the conjugation mode around the atom. For example, for the nineteen C atoms, there are three atomic charge distribution phenomena: (1) in the two phenyl rings and one thienyl ring, because C atoms have higher electronegativity than H atoms, thirteen C atoms conjugated with H atoms have negative atomic charges; (2) since the electronegativity of C is smaller than that of N, four C atoms joined with N atoms have positive atomic charges; (3) for the C(4) and C(10) atoms, they are indirectly conjugated with the N atoms, which makes them have negative atomic charges. In addition, for three N atoms, N(1) and N(2) atoms are connected with C atoms, so they both carry negative atomic charges. However, for the N(3) atom, there are two O atoms and one C atom bond with it, which leads the N(3) atom to have a positive charge. It is remarkable that, although the S atom has higher electronegativity than the C atoms, in the thienyl ring, the atomic charge values of S are positive, indicating that this S atom loses the capacity to coordinate with metallic ions, which is very different from that in a free thiophene molecule. On the whole, considering the atomic charge distributions and the steric effect, the title compound has three sites, namely N(2), O(1) and O(3), which have negative charge values and allow it to act as a multi-dentate ligand to coordinate with metallic ions.

### 2.4. Calculations of Nonlinear Optical Property

Since 2-pyrazoline derivatives have non-linear optical (NLO) properties [29], so we also made a prediction on the title compound. The predicted results were compared with the traditional NLO materials of urea and *para*-nitroaniline (PNA). On the basis of the MNDO Hamiltonian and PM3 parametrization with the MOPAC program package, the molecular hyperpolarizability value, *β_μ_*, the vector components along the dipole moment direction, of the title compound were calculated to be 4.997 × 10^−3^^0^ esu, which is greater than the value of urea (0.14 × 10^−3^^0^ esu calculated using the same method) [30] and smaller than that of PNA (6.801 × 10^−3^^0^ esu calculated using the same method). The comparisons indicate that the title compound is a potential NLO material. 

### 2.5. Electronic Absorption Spectra

**Figure 3:**
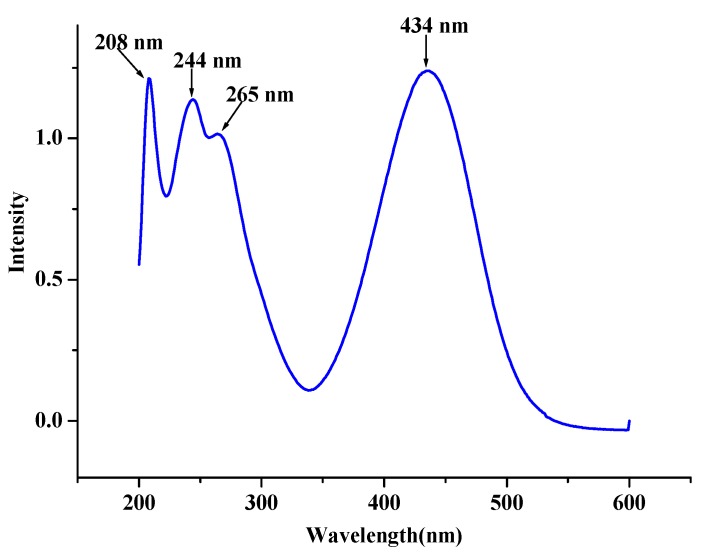
Electronic absorption spectra of the title compound measured in EtOH solution.

The electronic absorption spectrum of the title compound have been measured in EtOH solution and is shown in Figure 3. From Figure 3, one can find that there are four electronic transition peaks and three of them locate at about 208, 244 and 265 nm, respectively, which are in the ultraviolet region range. The fourth peak is at 434 nm, indicating the title compound solution has absorbed the blue-light and led to the solution being yellow.

**Figure 4:**
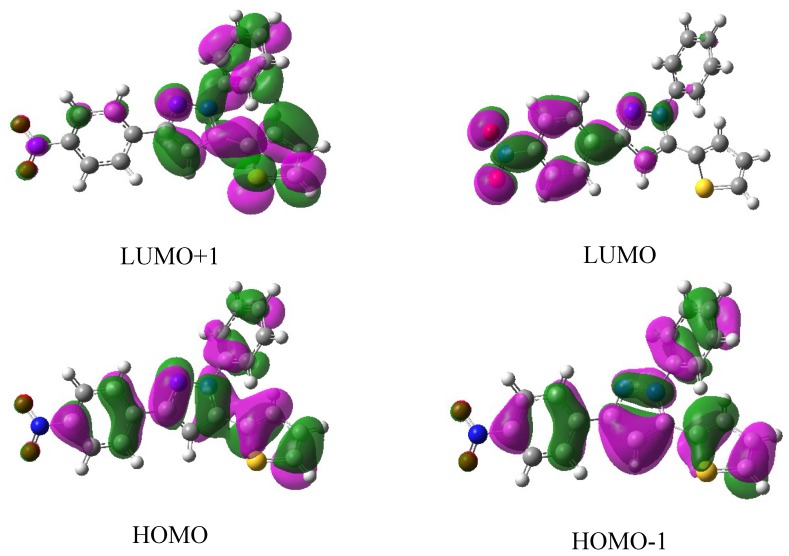
Some frontier molecular orbital stereographs for the title compound.

Natural population analyses based on the B3LYP/6-311G** optimized geometry show that the frontier molecular orbitals of the title compound are mainly composed of *p* atomic orbitals, so electronic transitions corresponding to above electronic spectra are mainly assigned to *n* → π* and *π* → π* electronic transitions. Figure 4 shows the surfaces of the HOMO-1, HOMO, LUMO and LUMO+1 for the title compound. As seen in Figure 4, when electron transitions take place, electrons are mainly transferred among the phenyl ring, nitrophenyl ring and thienyl ring, they correspond to the *n* → π* and *π* → π* electronic transitions.

### 2.6. Fluorescence Spectra

**Figure 5:**
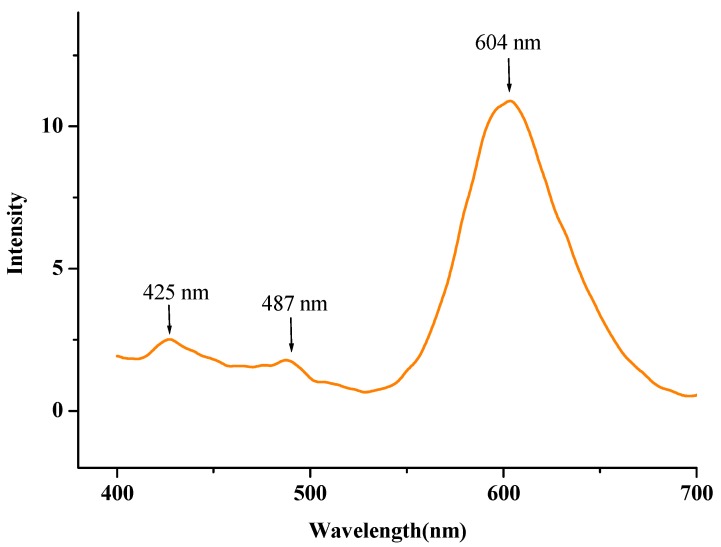
Solid-state fluorescence spectra of the title compound.

The solid-state fluorescence spectrum of the title compound is shown in Figure 5. The spectrum exhibits two weak emissions at 425 and 487 nm, respectively, which are in the violet-light region. A maximum emission band is at 604 nm, which is in the orange-light region. This result suggests the title compound is a potential orange-light emitting material.

## 3. Experimental and Theoretical Methods

### 3.1. Physical Measurements

Elemental analyses for carbon, hydrogen and nitrogen were performed by a Perkin-Elmer 240C elemental instrument. IR spectra (4000–400 cm^−1^) were recorded on a Nicolet FT-IR spectrophotometer as KBr pellets. Electronic absorption spectra were measured on a Shimadzu UV3100 instrument in EtOH solution and solid-state fluorescence spectra were measured on a F96-fluorospectrophotometer.

### 3.2. Synthesis

All chemicals were obtained from a commercial source and used without further purification. 1-(4-Nitrophenyl)-3-(2-thienyl)-2-propenyl-1-ketone (0.01 mol) and phenylhydrazine (0.015 mol) were mixed in acetic acid (40 mL) and stirred during reflux for 6 h. Then, the mixture was poured into ice-water to afford light-yellow solids. The solids were filtered and washed with water until the pH of the solution was about 7. Finally, the light-yellow solid crystals of the title compound were dried at room temperature. Yield 78.6%. Mp. 168.1–169.2. IR: *v* 3433(m), 3055(m), 1588(vs), 1550(s), 1492(vs), 1390(s), 1338(vs), 1267(m), 1127(s), 1107(s), 1062(m), 1034(w), 992(m), 839(s), 742(vs), 710(m), 685(s), 512(w) cm^−1^. Found: C, 65.51; H, 3.89; N, 12.23%. Calc. for C_19_H_13_N_3_O_2_S: C, 65.69, H, 3.77, N, 12.10%.

### 3.3. Crystallographic Study

The selected crystal of the title compound was mounted on an Rigaku raxis Rapid IP Area Detector diffractometer. Reflection data were measured at 293(2) K using graphite monochromated Mo-K*α* (*λ* = 0.71073 Å) radiation and a *ω*scan mode. The correction for *Lp* factors and empirical absorption were applied to the data. The structures were solved by direct methods and refined by full-matrix least-squares method on *F*_obs_^2^ using the SHELXTL software package [31]. All non-H atoms were anisotropically refined. The hydrogen atom positions were fixed geometrically at calculated distances and allowed to ride on the parent C atoms. The final least-square cycle gave *R* = 0.0707, *wR*_2_ = 0.1922. Atomic scattering factors and anomalous dispersion corrections were taken from International Table for X-ray Crystallography [32]. A summary of the key crystallographic information is given in Table 4.

CCDC-990511 contains the supplementary crystallographic data for this paper. These data can be obtained free of charge at www.ccdc.cam.ac.uk/conts/retrieving.html (or from the Cambridge Crystallographic Data Centre (CCDC), 12 Union Road, Cambridge CB2 1EZ, UK; Fax: +44(0)1222-336033; E-Mail: deposit@ccdc.cam.ac.uk).

### 3.4. Computational Methods

Initial molecular geometry of the title compound was taken from its crystal structure. Then, DFT calculations at B3LYP/6-311G** levelof theory by the Berny method [33] were performed with the Gaussian 03 software package [34]. Vibrational frequencies calculated ascertain the structure was stable (no imaginary frequencies). Natural Bond Orbital (NBO) analyses were also performed on the optimized structure. On the basis of the MNDO Hamiltonian [35] and PM3 parametrization [36] with the MOPAC [37] program package, the molecular hyperpolarizability value was also calculated. All calculations were performed on a DELL PE 2850 server and a Pentium IV computer using the default convergence criteria.

**Table 4:** Summary of crystallographic results for the title compound.

Empirical Formula	C_19_H_13_N_3_O_2_S
Formula weight	347.38
Temperature	293(2) K
Wavelength	0.71073 Å
Crystal system, space group	Monoclinic, *C*2/*c*
Unit cell dimensions	*a* = 28.023(7) Å
	*b* = 7.8005(15) Å *β* = 122.69(2)°
	*c* = 18.008(7) Å
Volume	3312.9(17) Å^3^
*Z*, Calculated density	8, 1.393 Mg/m^3^
Absorption coefficient	0.213 mm^−1^
*F*(000)	1440
*θ* range for data collection	3.26 to 25.00°
Limiting indices	−33 ≤ *h* ≤ 33, −9 ≤ *k* ≤ 8, −21 ≤ *l* ≤ 21
Reflections collected/unique	10,658/2887 [*R*_int_ = 0.0476 ]
Refinement method	Full-matrix least-squares on *F*^2^
Data/restraints/parameters	2887/0/226
Goodness-of-fit on *F*^2^	1.120
Final *R* indices [*I* > 2*σ* (*I*)]	*R*_1_ = 0.0707, *wR*_2_ = 0.1922
*R* indices (all data)	*R*_1_ = 0.0911, *wR*_2_ = 0.2071
Largest diff. peak and hole	0.402 and −0.404 e. Å^−3^

## 4. Conclusions

1-Phenyl-3-(4-nitrophenyl)-5-(2-thienyl)-2-pyrazoline has been synthesized and characterized, by a variety of methods including IR, X-ray single crystal diffraction, UV-Vis and fluorescence spectroscopy. For the title compound, DFT calculations at B3LYP/6-311G** of the structure and NPA atomic charge distributions have been carried out. Experimental and predicted results indicate that the optimized geometry at B3LYP/6-311G** level can represent the molecular structure. NPA atomic charge distributions show the title compound may be acted as multi-dentate ligand to coordinate with metallic ions. The electronic absorption spectra measurement of the title compound exhibits four absorption bands and DFT calculations indicate the above electronic spectra are mainly assigned to *n* → π* and *π* → π* electronic transitions. The solid-state fluorescence spectrum reveals the title compound is a potential orange-light emitting material.
